# A scoping review of the role of HIV-related stigma and discrimination in noncommunicable disease care

**DOI:** 10.1371/journal.pone.0199602

**Published:** 2018-06-21

**Authors:** Melissa A. Stockton, Kayla Giger, Laura Nyblade

**Affiliations:** Global Health Division, International Development Group, RTI International, Washington, DC, United States of America; The University of Warwick, UNITED KINGDOM

## Abstract

**Background:**

People living with HIV are increasingly burdened by noncommunicable diseases (NCDs) as a result of the NCD susceptibility that accompanies increased life expectancy and the rising global prevalence of NCDs. Health systems are being strengthened and programs are being developed to address this burden, often building on HIV care strategies and infrastructure or through integrated care models. HIV remains a stigmatized condition and the role of HIV stigma in the provision of NCD care is not well understood.

**Methods:**

We conducted a scoping literature review of both peer reviewed and grey literature to identify evidence of the role of HIV stigma in the NCD-care continuum (prevention, diagnosis, care seeking, retention in care, and adherence to treatment of NCDs). We searched PsychInfo and Pubmed and conducted additional searches of programmatic reports and conference abstracts. Included studies were published in English within the past decade and examined HIV-related stigma as it relates to NCD-care or to integrated NCD-and HIV-care programs.

**Results:**

Sixteen articles met the inclusion criteria. Findings suggest: fear of disclosure, internalized shame and embarrassment, and negative past experiences with or negative perceptions of health care providers negatively influence engagement with NCD care; HIV stigma can adversely affect not only people living with HIV in need of NCD care, but all NCD patients; some NCDs are stigmatized in their own right or because of their association with HIV; integrating NCD and HIV care can both reduce stigma for people living with HIV and a present a barrier to access for NCD care.

**Conclusion:**

Due to the dearth of available research and the variability in initial findings, further research on the role of HIV stigma in the NCD-care continuum for people living with HIV is necessary. Lessons from the field of HIV-stigma research can serve as a guide for these efforts.

## Introduction

People living with human immunodeficiency virus (PLHIV) across the globe now live longer due to the improvements in and expanded access to antiretroviral therapy (ART) [1–4]. Along with increased life expectancy comes an increased burden of noncommunicable diseases (NCDs) [5]. In addition, PLHIV are at increased risk for select NCDs due to the nature of HIV and side effects of its treatment [6–7] such as serious non-acquired immune deficiency syndrome (AIDS) events including lymphoma, cervical cancer, cardiovascular disease, hypertension, depression and neurological disorders, among others [8–11]. Finally, the prevalence of NCDs in low- and middle-income countries (LMICs), many of which have a high burden of HIV, are rising. This is due in part to increases in rates of smoking, unhealthy eating, harmful use of alcohol, and inactivity and the associated increases in rates of obesity and high blood pressure [12,13], all of which also affect PLHIV [13], as seen in other places across the globe. The combined effect of increased life expectancy, NCD susceptibility and global epidemiological trends has produced a substantial burden of NCDs among PLHIV in LMICs. Consequently, PLHIV may now need to venture beyond specialized HIV clinics and engage more regularly with the broader health system to obtain the care they need [14,15].

Given the growing burden of NCDs in both the general population and among PLHIV, health care systems must be strengthened to provide needed NCD services. To meet this need, policy makers are beginning to draw from the extensive investment in strategies and systems that support lifelong HIV care and the lessons learned from HIV treatment scale-up [16–18]. Programs to provide NCD care as part of primary or chronic-disease care—such as for HIV—are being developed across the globe, but particularly in LMICs [19], where HIV-care infrastructure is now being leveraged to meet NCD health care needs of both PLHIV and the general population [16, 20, 21]. For example, NCD care may be offered as part of the provision of HIV care, as seen in depression treatment integrated into HIV care in Cameroon [22] or cervical cancer screening offered to women enrolled in HIV care in Kenya [23]. Or, chronic disease-care programs may be developed out of existing HIV-care programs that utilize HIV-care facilities and human resources to provide care for both HIV and NCDs, as seen in the fully integrated HIV-NCD care pilot program that is leveraging HIV resources to provide care for both PLHIV and NCD patients in the same health clinics in Malawi [24]. As more health systems look to integrated models of care for NCDs that build on HIV infrastructure, the same factors that influence the uptake and provision of HIV care may also impact NCD care.

One key factor that negatively impacts care is HIV-related stigma and discrimination. Stigma and discrimination persist in health care delivery systems across the globe, violate human rights and hinder access and engagement in the HIV-care continuum [25–29]. PLHIV may fear both being identified as someone living with HIV, and the poor treatment that might come from such identification, and thus avoid accessing services. Then, once in treatment, PLHIV may experience a range of manifestations of stigma from verbal abuse to neglect, denial of care, and involuntary disclosure of their sero-status [29]. As PLHIV seek care outside their regular HIV-care settings, there is some evidence that suggests the risk of encountering stigma related to HIV within the health system may rise [30, 31]. For example, a survey of dentists in Central India found that only 39.2% of dentist were willing to treat PLHIV [30]. Such observations suggest HIV stigma may also impact the NCD-care continuum, from prevention to diagnosis through retention and adherence to treatment. Furthermore, as health care systems evolve to meet the growing demands associated with NCDs, there is potential for HIV stigma to shape NCD service delivery. For example, HIV-stigma may influence where and how NCD care is provided both for PLHIV and for HIV-seronegative individuals [16]. For example, in Zambia, a program linking cervical cancer screening and HIV care placed screening clinics in the same health center near to, but not directly within, the HIV clinic, noting that concerns around HIV-related stigma could prevent HIV-seronegative women or those unaware of their HIV status from accessing cervical cancer screening [32, 33].

Consequently, the growing number of people in need of NCD care, the increased susceptibility of PLHIV to NCDs, and the persistence of HIV stigma as barriers to care, all point to the potential for HIV stigma to undermine the NCD-care continuum. In order to strengthen efforts to reduce NCD risk and improve NCD care in general and for PLHIV in particular, an improved understanding about whether and how HIV stigma impact NCD diagnosis and care is needed [11]. Thus, the purpose of this scoping review was to identify what is known about the role of HIV stigma as it relates to NCD-care across the globe. In line with the aim of our research, a scoping review of literature was chosen as the most appropriate methodology as it would allow us to rapidly map all types of available evidence underpinning this research topic [34].

## Methods

We conducted a scoping literature review to identify existing evidence of the role of HIV stigma on the NCD-care continuum. Our objectives were to document the available evidence describing the role of HIV-related stigma on any aspect of the continuum of care for NCDs and identify the emerging themes related to HIV-stigma and either NCD care or the integration of NCD and HIV care. The “continuum of care” was defined as prevention, diagnosis, care seeking, retention in care, and adherence to treatment. NCDs were limited to the priority NCDs under the Sustainable Development Goal 3: cancer, cardiovascular disease, chronic pulmonary disease, diabetes, anxiety, and depression [35] We present the methodology below according to the Arskey and O’Malley framework [34]. This framework provides an approach to conducting a scoping review through (1) identifying the research question; (2) identifying relevant studies; (3) study selection; (4) charting the data; and (5) collating, summarizing and reporting the results.

### Identifying relevant studies

We conducted a broad search of peer-reviewed, grey (programmatic) literature and conference abstracts. We searched PsychInfo and Pubmed to identify peer-reviewed literature. Additionally, we conducted targeted searches of grey literature databases such as the US Centers for Disease Control and Prevention Stacks, US Agency for International Development’s Development Experience Clearinghouse, and conference abstracts from the 21^st^ International AIDS Conference (AIDS 2016) and the 9^th^ International AIDS Society (IAS) Conference on HIV Science (IAS 2017). We used the following search terms were used to identify relevant literature: “HIV” paired with either “stigma,” “discrimination,” or “attitudes” in addition to “hypertension,” “cardiovascular disease,” “diabetes,” “chronic respiratory,” “chronic pulmonary disease,” “cancer,” “depression,” “anxiety,” or “mental health.” See the supplementary materials for an example of the full search strategy.

From the search, the identified peer-reviewed citations and abstracts were uploaded, organized and reviewed using Covidence (a systematic review software) from their respective databases Two reviewers (MAS and KG) independently screened titles and abstracts for eligibility. These same two reviewers screened grey literature within their respective databases. The senior author (LN) discussed any discrepant assessments and resolved any outstanding disputes.

### Study selection

We included articles describing: (1) research measuring HIV-related stigma and its association with or effect on the continuum of care for NCDs; (2) research exploring HIV-related stigma as it relates to the continuum of care for NCDs; (3) programs or interventions addressing HIV stigma towards PLHIV seeking care for NCDs; (4) integrated NCD- and HIV- care programs that examine HIV stigma; or (5) reviews that provide insight on the aforementioned topics. In an effort to capture all available evidence, we did not restrict the study geography or methodology. Additionally, we only included articles published in the last 10 years prior to May 30, 2017 and written in English. However, we excluded studies if they examined the association between HIV stigma and risk factors for NCDs (e.g., weight or smoking) or exclusively examined stigma related to NCDs (e.g., mental health stigma).

### Charting data

We exported the title, author, journal and year of publication for studies that met the inclusion criteria into an excel spreadsheet for data abstraction and charting. The same two authors independently abstracted data on the following topics from each included article: country, NCD examined, study type and design, population, NCD outcomes examined, type of stigma(s) measured or examined, and key findings. We then organized key findings into the following categories: the role of HIV stigma in NCD care; the role of stigma related to NCDs and other factors in NCD care; inter-related stigmas; and stigma as it relates to integration of NCD and HIV care. Abstracted data were reviewed for consistency, and the senior author (LN) discussed any discrepancies and settled any disputes.

Additionally, the two reviewers appraised all included articles using the Joanna Briggs Institute tools recommended for mixed method reviews synthesizing at least two types of data [36–39]. The studies were all scored using appropriate checklists; qualitative studies were scored out of 10 questions, the cross-sectional and longitudinal were scored out of eight questions and the randomized control trial was scored out of 12 questions. This appraisal allowed the methodological quality of included studies to be assessed; the possibility of bias in the design, conduct, and analysis to be considered; and the strength of the evidence to be further evaluated.

## Results

**Fig 1:**
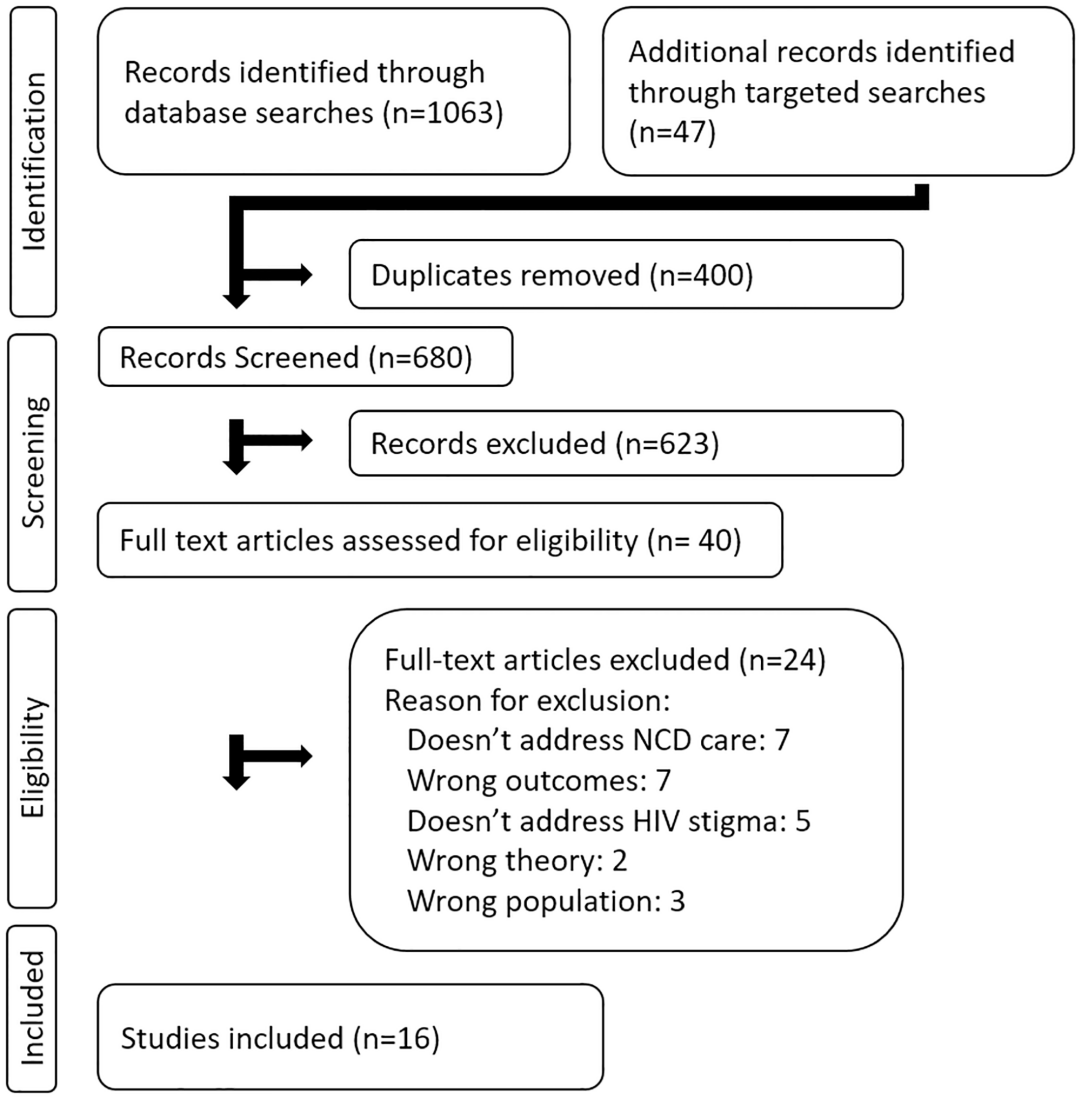
PRIMSA flowchart.

We identified 663 peer-reviewed abstracts and 40 were advanced to a full-text review. Of the 40 articles that underwent full-text review, 16 met the inclusion criteria. (Fig 1) None of the grey literature met the inclusion criteria. The results section first presents the overall characteristics of the included studies and then describes the findings that emerged under four overarching themes: HIV stigma and NCD care; stigma related to NCDs; intersectional stigma; and integration of NCD and HIV care.

### Study characteristics

The review yielded 16 articles and 15 distinct studies (Table 1). Of note, both of the articles by Rosser et al. present findings from the same randomized control trial (RCT); Rosser et al. 2016 [40] presents cross-sectional analysis of their baseline data, while Rosser et al. 2015 [41] presents the longitudinal analysis of the completed RCT. The studies include a range of methods, with the majority being qualitative or mixed methods (n = 9). Of these nine qualitative and mixed method studies, seven conducted focus group discussions and in-depth interviews, and two separate studies conducted literature reviews and key informant interviews. Of the seven quantitative studies, five were cross-sectional studies, one RCT, and one longitudinal study. Twelve studies collected and analyzed primary data, three studies evaluated integrated NCD/HIV programs, and one evaluated the effect of receiving a health talk on uptake of cervical cancer screening. Most studies were conducted in Africa (n = 7) and the United States (n = 5). One study each was conducted in Cambodia, and India. One of the two mixed-method literature review studies conducted interviews with key stakeholders in the United Kingdom, while the other interviewed experts from across the globe. Included studies ranged in size and scope; one study surveyed 145 women living with HIV on cervical cancer screening [42], while another study used longitudinal clinical data from patients living with HIV (4,793), diabetes (2,638) hypertension(1,419 hypertensive), and other chronic diseases (299) to evaluate a program integrating HIV and chronic disease care [43]. Participants in the articles included in this review included patients living with HIV, NCD patients, providers (both HIV specialists and non-HIV specialists), and policy makers. NCDs investigated in the identified studies included cancer, diabetes, cardiovascular diseases, and depression. No studies were identified that examined care for chronic pulmonary disease, and all seven of the cancer studies examined cervical cancer.

**Table 1:** Characteristics of included studies (n = 16).

**Study Type**	
Qualitative	7
Qualitative + Literature Review	2
Cross Sectional^a^	5
Randomized Control Trial	1
Longitudinal	1
**Noncommunicable Disease Investigated**	
Cancer	7
Depression	3
Cardiovascular Disease	1
Multiple^b^	5
**World Health Organization Region**	
Africa	7
Americas	5
Europe	1
South-East Asia	1
Western Pacific	1
Multiple	1

^a^Two articles present findings from the same randomized control trial. However, one article analyzes the unstratified baseline data for the trial and is thus categorized as a cross-sectional study.

^b^Includes cardiovascular disease, diabetes, mental health, and general noncommunicable diseases.

None of the included articles were excluded following appraisal with the quality assessment tools. The qualitative studies’ scores ranged between 6 and 8 out of 10, the randomized control trial scored 9 out of 12, and the cross-sectional and longitudinal studies’ scores ranged between 4 and 8 out of 8. (Table 2) Due to the methodological heterogeneity of the included studies, a comprehensive meta-analysis for all outcomes was not possible.

**Table 2:** Critical appraisal results.

**Qualitative Checklist**	
Ameh et al. 2017	8/10
Andrasik et al. 2008	8/10
Duffy et al. 2017	6/10
Hutchinson et al. 2016	6/10
Kenya et al. 2015	8/10
Kumakech et al. 2014	7/10
Le et al. 2016	7/10
Venables et al. 2016	7/10
White et al. 2012	8/10
**Cross-Sectional Checklist**	
Bynum et al. 2016	6/8
Chan et al. 2016	5/8
Janssens et al. 2007^a^	4/8
Kamitani et al. 2015	8/8
Moore et al. 2017	4/8
Rosser et al. 2016	7/8
**Randomized Control Trials Checklist**	
Rosser et al. 2015	9/12

^a^This longitudinal study was appraised with the cross-sectional checklist.

Four studies used or adapted established stigma measures [40–42, 44]. Two studies used alternative quantitative methods to assess HIV stigma [43, 45]. Moore et al. [45] measured HIV stigma using both a dichotomous item that asked directly about HIV stigma and an open-ended question on barriers to attending group therapy sessions. Janssens et al. [43] used longitudinal patient clinic data to assess case load and patient outcomes as means of commenting on the potential role of stigma in integrated NCD- and HIV-care programs. For more detailed information on the included study characteristics see Table 3.

**Table 3:** Included studies.

Author	Country	NCD	Population	Study Design and Overview
Andrasik et al. 2008	United States	Cervical cancer	35 African American women living with HIV aged 18–49 without screening for cervical cancer screening in the past 5 years and a previous hysterectomy	Exploratory, Qualitative—*In-depth interviews on perceived barriers to cervical cancer screening*
Ameh et al. 2017	South Africa	Noncommunicable diseases (NCDs) including hypertension and diabetes	61 diabetic or hypertensive patients age ≥ 18, 7 operational managers, 1 sub-district health manager	Evaluation, Qualitative—*Focus group discussions and in-depth interviews used to evaluate the integrated chronic disease management (ICDM) model—a diagonal approach to health system strengthening that integrated the vertical HIV program with horizontal general health system and had both health facility and community/population components—that focused on experiences interacting with providers*, *the quality of integrated chronic care*, *and policy environment for implementing the ICDM model*.
Bynum et al. 2016	United States	Cervical cancer	145 women living with HIV aged ≥ 18 seeking health care from public clinics and social services from community-based AIDS Service Organizations	Exploratory, Cross-sectional—*Surveyed women on cervical cancer prevention knowledge*, *attitudes*, *beliefs*, *behaviors*, *and social and structural factors associated with care engagement*.
Chan et al. 2016	India	Depression	69 HIV clinicians	Exploratory, Cross-sectional—*Surveyed clinicians asking for level of agreement with statements on the etiology*, *importance*, *and management of depression among people living with HIV (PLHIV) and whether they felt capable and willing to manage depression in PLHIV*.
Duffy et al. 2017	Multiple	NCDs	5 authors of articles identified in literature review considered to be leaders of integrated NCD and HIV programs	Exploratory, Literature Review + Qualitative—*Literature review and key-informant interviews on existing models of NCD and HIV care and treatment service integration in low-income countries; implementation challenges and suggestions to improve integrated service delivery*.
Hutchinson et al. 2016	United Kingdom	NCDs including cardiovascular disease (CVD) and mental health	2 HIV specialists, 2 HIV specialist general practitioners, 1 general practitioner with a special interest in HIV, and 5 general practitioners	Exploratory, Literature review + Qualitative—*Literature review and key-informant interviews focused on understanding contemporary models of shared primary and specialist care of PLHIV*, *including barriers to implementation and factors influencing their success*, *to inform future HIV care provision in the United Kingdom*.
Janssens et al. 2007	Cambodia	Diabetes, hypertension, other chronic diseases	4,793 patients living with HIV, 2,638 with diabetes, 1,419 with hypertension, and 299 with other chronic diseases	Evaluation, Longitudinal—*Evaluated a program offering integrated care for HIV/AIDS*, *diabetes*, *and hypertension within a chronic disease clinic setting using longitudinal data to assess case load and patient outcomes*.
Kamitani et al. 2015	United States	CVD	67 individuals who self-identified as living with HIV and Asian, aged at least 18 years	Exploratory, Cross-sectional—*Surveyed individuals on knowledge*, *self-efficacy*, *and self-perceived risk regards CVD and acute coronary syndrome*.
Kenya et al. 2015	United States	Cervical cancer	21 Haitian women living with HIV, aged 30–60, who had not had a hysterectomy	Exploratory, Qualitative—*Focus group discussions on human papilloma virus (HPV)/cervical cancer knowledge*, *HIV issues associated with HPV screening*, *HPV/cervical cancer screening behaviors*, *HPV/cervical cancer research targeting women living with HIV*, *methodological preferences for an effective HPV/cervical cancer education*.
Kumakech et al. 2014	Uganda	Cervical cancer	16 providers and policy makers with experience in the delivery of HIV and cervical cancer screening services	Exploratory, Qualitative—*In-depth interviews on perceptions of providers and policy makers toward the integration of HIV and cervical cancer screening services*.
Le et al. 2016	United States	Depression	7 providers and staff with training in medicine and social work	Exploratory, Qualitative—*Focus group discussion on adapting an effective depression screening tool for low-income African American urban Washington*, *DC*, *residents living with HIV that focused on experiences and current mental health needs of African American patients living with HIV that are shaped by socio-ecological factors*.
Moore et al. 2017	United States	Depression	150 veterans living with HIV aged ≥ 50 years receiving HIV-related medical care	Exploratory, Cross-sectional—*Surveyed individuals on mental health needs and barriers to participating in mental health treatment*.
Rosser et al. 2016	Kenya	Cervical cancer	419 women (55% living with HIV) aged 23–64 eligible for cervical cancer screen, but had not previously been screened	Exploratory, Cross-sectional^a^—*Surveyed women on HIV stigma*, *cervical cancer stigma*, *and cervical cancer screening acceptability*.
Rosser et al. 2015	Kenya	Cervical cancer	207 intervention and 212 control women (55% living with HIV) aged 23–64 eligible for cervical cancer screen, who had not previously been screened	Evaluation, RCT^a^—*Randomized women to receive a brief health talk on cervical cancer which included a guided discussion about barriers to screening and fears or stigma associated with screening or to be controls*. *Participants surveyed at enrollment (all)*, *immediately after the talk (intervention arm)*, *and at three months follow-up (all) on HIV-stigma and cervical cancer knowledge*, *screening behavior*, *screening awareness*, *perceived risk*, *acceptability*, *and stigma*.
Venables et al. 2016	Kenya	Hypertension and diabetes	25 HIV, diabetes, or hypertension health care providers, 42 medication adherence club (MAC) and 39 non-MAC HIV, diabetes, or hypertension patients	Evaluation, Qualitative.—*Focus group discussion*, *in-depth interviews*, *and participant observation to evaluate MACs for individuals in need of chronic care that focused on people’s experiences of MACs*, *the challenges they faced*, *and their perceptions about models of care for chronic conditions*.
White et al. 2012	Zambia	Cervical cancer	60 women (1/3 living with HIV) present at clinic, aged 18–49, eligible for screening, willing to undergo a pelvic examination	Exploratory, Qualitative.—*Focus group discussions and in-depth interviews to elicit detailed perceptions of cervical cancer held by Zambian women newly screened for cervical cancer*.

^a^Two articles present findings from the same randomized control trial. However, one article analyzes the unstratified baseline data for the trial and is thus categorized as a cross-sectional study

### Emerging theme one: HIV stigma and the NCD care continuum

The review revealed several studies (n = 15) that examined the role of HIV stigma along the NCD-care continuum; six studies examined prevention; three studies examined diagnosis and treatment; two studies examined general care seeking and two studies examined the provision of care. No studies examined the role of HIV stigma as part of treatment adherence or retention in NCD care. Of the included studies, we identified only one intervention that included specific stigma-reduction efforts [41] as part of NCD service delivery. Based on the available evidence identified in this review, these studies had mixed conclusions about the relationship between different types HIV stigma and engagement in NCD care by PLHIV.

All of the studies (n = 6) that examined HIV stigma and NCD prevention examined cervical cancer screening [40–42, 46–48]. The three qualitative studies identified HIV stigma as a barrier to screening [46–48]. One study, including both women living with and without HIV, described how women fail to screen for fear of the diagnoses’ association with HIV [48], and two others described how shame, embarrassment, negative past experiences, or fear of being mistreated by health care providers deters women living with HIV from screening [46, 47]. However, one cross-sectional study in the United States [42] and the articles presenting findings from the baseline and endline analysis of an RCT in Kenya found that HIV stigma was not significantly associated with or predictive of cervical cancer screening for women living with and without HIV [40,41]. While these findings that screening and stigma are not associated seem to contradict others’ findings, the authors of the cross-sectional study conducted in the United states noted that their measures used to assess HIV stigma and health care discrimination may not have fully captured the complex constructs [42].

Three studies examined the role of HIV-stigma in NCD diagnosis and treatment, and all focused on identifying and managing depression [45, 49, 50]. One small qualitative study examined the adaptation of an effective depression screening tool for low-income African Americans living with HIV. While this study included only seven providers and staff with training in medicine and social work, it found that HIV stigma interferes with clinical efforts to identify individuals struggling with depression [49]. In a cross-sectional study in India with health providers, when asked about their attitudes towards PLHIV with depression, 62% of respondents were either neutral or agreed with the statement “depression is a sign of personal weakness,” and 53% of respondents felt that it was difficult to work with depressed PLHIV [50]. A cross-sectional study on barriers to mental health treatment among veterans living with HIV in the United States found that among those with positive Patient Health Questionnaire-2 screens, participants who reported experiencing HIV stigma were more likely to be in treatment (χ2 = 4.22, P = .04) compared to those who did not report HIV stigma. However, among all participants, patient-reported barriers to mental health treatment included HIV stigma, shame, disinterest, and other health problems (< 18% frequency).

Two studies examined HIV stigma and general care seeking for NCDs [44, 51]. A study of developing models of shared primary and specialist care for PLHIV in need of NCD care recognized the role of stigma in hindering disclosure and complicating the provision of routine care for primary care providers [51]. A cross-sectional study that investigated HIV stigma and cardiovascular disease among Asian Americans living with HIV found HIV stigma was negatively correlated with self-efficacy in recognizing and seeking medical attention for a heart attack (r = -0.43, p, .0005) [44].

Only two studies examined HIV-stigma reduction as part of the provision of NCD care to PLHIV [41, 51]. The one included RCT evaluated the provision of a brief health talk on cervical cancer that included a guided discussion about barriers to screening, such as fear of stigma. This study measured perceived HIV-related stigma among both women living with and without HIV. They found that HIV-stigma scores decreased significantly more among those who received the health talk than among those who did not receive the health talk (Z = 2.0, p = 0.05), but screening uptake did not significantly differ between groups [41]. Hutchinson et al. described models of shared primary and specialist care for PLHIV, barriers to implementation of the shared models, and factors influencing their success. The Hutchinson et al. study used findings from a literature review of care in high-income settings and interviews with key stakeholders—HIV specialists, general practitioners in shared care, and key representatives from the British HIV Association and HIV commissioners—in the United Kingdom. The study found that fear of sero-status disclosure due to HIV stigma was a barrier to quality care. It identified the following strategies to manage concerns around disclosure and develop “HIV-friendly” practices: 1) involve the whole general practice team, including non-clinical staff such as receptionists; 2) ensure appropriate disclosure within a practice (e.g., reception staff having access to records for appropriate administration only); 3) avoid inadvertent disclosures due to audible conversations at reception or visible diagnoses on computer screens; and 4) reassure patients by, for example, displaying notices about confidentiality in general practices [51].

### Emerging theme two: Stigma related to NCDs

NCDs may carry their own stigma. Seven articles discussed stigma related to cervical cancer [40, 41, 48, 52], depression [49, 50], and diabetes and hypertension [53] alongside HIV-related stigma. For example, Rosser et al. (2015 and 2016) presented analyses from different timepoints in their evaluation of a cervical cancer screening intervention, measuring both cervical cancer-related and HIV-related anticipated stigma (e.g., fear of being treated badly by health workers or becoming a social outcast should others find out they had cervical cancer). At baseline, anticipated cervical cancer stigma was 13.7% among women living with HIV and 28.5% among HIV-negative women, lower than anticipated HIV stigma which was 23.2% among women living with HIV and 39.3% among HIV-negative women [40]. Le et al.’s study conducted with providers on the provision of depression care for PLHIV found that providers recognized that social stigma was associated not only with HIV, but also with depression [49]. Another study evaluated the use of medication adherence clubs—groups that meet to receive medication refills and engage in health discussions—for patients with HIV, diabetes, or hypertension. This study found that while concerns around stigma and disclosure tended to focus on HIV patients, diabetes and hypertension patients also brought up the challenges in disclosing their chronic disease with others [53].

### Emerging theme three: Intersectional stigma

Individuals may also face discrimination that influences their health for reasons unrelated to their health status and belonging to multiple stigmatized groups has been shown to compound the negative effects of stigma, though the mechanisms and degree to which this occurs is not well understood [54]. While none of the identified studies unpacked intersectional stigma, whether between disease stigma and other stigmas, or between multiple disease stigmas, three studies recognized that HIV stigma may interact with stigma related to NCDs. Rosser et al.’s cross-sectional cervical cancer study found a high correlation between cervical cancer- and HIV-stigma scores (correlation coefficient 0.72) and found that HIV-positivity was a significant predictor of both lower anticipated cervical cancer stigma and lower anticipated HIV stigma [40]. A qualitative study of attitudes and beliefs around cervical cancer found that cervical cancer is highly stigmatized in Zambia, not only because of its anatomic location, dire natural course, and connections to socially condemned behaviors, but also because of its association with HIV [48]. In light of both the stigma attached to HIV and widespread stigmatizing views of depression among non-psychiatric clinicians, Chan et al. conducted a cross-sectional survey that measured the intersection of depression and HIV-related stigma among HIV-care providers by measuring stigmatizing attitudes toward PLHIV with depression [50]. In this sense, Chan et al. measured compounded depression and HIV stigma, without separately measuring either HIV stigma or depression stigma.

Only one study discussed stigma or discrimination not specifically related to a health condition. This study on cervical cancer screening among African Americans living with HIV recognized the potential role of racial discrimination. This study found that, in addition to the detrimental effects of the perception that medical providers hold negatives attitudes towards PLHIV, believing providers hold negative attitudes towards racial and ethnic minority groups also hindered access to cervical cancer screening [46].

### Emerging theme four: Integration of NCD and HIV care

As NCD- and HIV-care programs are increasingly being integrated into one another or into the primary health care system, this review included selection criteria to identify integrated programs that recognized the role of HIV stigma in seeking and delivering NCD care. Five such articles on integrated NCD- and HIV-care programs were identified. Of these articles, three evaluate existing integrated chronic disease programs that provide care to both PLHIV and NCD patients [43, 53, 55]; one explores providers’ and policy makers’ attitudes towards integrating cervical cancer and HIV treatment [56], and one discusses the strengths and weaknesses of various models for integrating NCD and HIV treatment [52].

Four studies found that HIV stigma would hinder access to care for HIV-negative NCD patients, as they may be reluctant to access care from programs that are integrated with HIV care [52, 53, 55, 56]. Ameh et al. presented a case study of seven primary health care facilities in South Africa implementing an integrated chronic disease management (ICDM) model. The ICDM model aims to improve NCD care by integrating vertical HIV programs into existing primary care infrastructure and by utilizing successful HIV program innovations to improve the quality of chronic disease care. For example, health facilities adopting the ICDM model borrowed strategies that had been successful in HIV programs and prioritized defaulter-tracing activities for NCD patients, established a community outreach team for community-oriented chronic care, and held population-level health promotion and screening initiatives. Through interviews with NCD patients and program managers, this study found that HIV stigma negatively impacted defaulter tracing and continued engagement in NCD care [55]. A qualitative study, conducted among health care providers and policy makers in Uganda, of integrating HIV testing and cervical cancer screening found that participants believed HIV stigma would be a major barrier to cervical cancer screening, as potential clients would be afraid of being required to test for HIV. The providers recommended that integration strategies make an effort to dissociate HIV testing from cervical cancer screening through educational materials that advertise opt-out testing options, but also aim to reduce HIV stigma [56].

Four studies examined the potential effects of various integrated models of care on HIV stigma, and all four concluded some level of integration could potentially result in HIV-stigma reduction for PLHIV [43, 52, 53, 55]. The study conducted in Cambodia that used longitudinal clinical data to evaluate an integrated chronic disease clinic justified integration in that "by providing care for seropositive clients and patients with other chronic diseases within the same facility, it was hoped that facility-related stigma could be reduced” for PLHIV. The authors conclude their evaluation by citing that the increasing inflow of both HIV and diabetic patients is indicative of stigma reduction and program acceptability [43]. Ameh et al.’s study on the ICDM model found that providers believe stigma for PLHIV was reduced, as patients seeking HIV care were no longer segregated and it was therefore more difficult to identify who was HIV positive [55]. A qualitative evaluation of medication-adherence clubs for HIV, diabetes, and hypertension patient care in Kenya found that combining HIV-negative NCD clients and clients living with HIV together reduced HIV stigma for PLHIV, as those living with HIV were not treated differently from people with diabetes or hypertension. However, Ameh et al. also noted that providers and PLHIV clients less familiar with the club were concerned that HIV patients’ sero-status would be revealed in these meetings, though this concern was not raised by club members themselves [53]. Duffey et al.’s mixed-methods study, which conducted a review of literature on different models of integrating HIV and NCD care and interviewed leaders of integrated NCD/HIV programs, found that NCD clients may fear being identified as HIV positive when seeking care from an NCD program that is physically integrated into an HIV health care setting. However, they also found that integrating HIV services into NCD services at the primary health care level or simultaneous introduction of an integrated program for HIV and NCD services may reduce stigma for PLHIV, given that services are physically located outside of HIV facilities [52].

## Discussion

The studies identified in this literature review, while few in number, provide some useful insights. In particular, findings suggest that fear of disclosure, internalized shame and embarrassment, and negative past experiences with or perceptions of health care providers as stigmatizing all negatively influence engagement with NCD care for PLHIV [46, 47, 50, 51, 53, 56]. As well, we found stigma, both related to HIV and to certain NCDs, may affect engagement in NCD care for all patients in need of services regardless of HIV status. Some of the included studies on integrated care suggest that programs leveraging existing HIV infrastructure and utilizing certain integrated models of care could be hindered by HIV stigma. For example, Ameh et al.’s case study of ICDM found that NCD patients did not welcome community-based outreach or defaulter-tracing as these are common HIV services and NCD patients were afraid people would assume there were living with HIV [55]. In addition, NCDs may be stigmatized in their own right and NCD-related stigma, particularly stigma related to cervical cancer, may increase as a result of its co-morbid association with HIV. Interestingly, none of the articles in this review unpacked compounding or intersectional stigma, though several studies explored how HIV stigma may interact with stigma related to cervical cancer and depression or suggested that facing multiple stigmas may further exacerbate barriers to care and worsen health outcomes [46, 49]. Some of the identified strategies for creating and maintaining a stigma-free environment included training the entire clinic staff (including non-clinical staff) and adapting practices to prevent involuntary disclosure [51]. As well, this literature review identified one intervention that nominally attempted HIV-stigma reduction as part of NCD care. This health-education intervention, while not aimed specifically at reducing stigma, did include a minimalist stigma-reduction component for both HIV- and cervical cancer-related stigma and was successful at reducing perceived stigma among potential cervical cancer screening clients, but did not affect screening uptake [41].

Previous research efforts compliment many of our findings. Other studies have shown that PLHIV may be more likely to either fear or actually encounter both inadvertent or intentional discrimination when accessing care in settings where providers are less familiar with HIV [57, 58]. Research has also demonstrated that HIV stigma may be a barrier to care for HIV-negative individuals, particularly for services that patients believe will require them to be tested for HIV, as seen from White et al.’s investigation of cervical cancer stigma [48] and other studies on antenatal care seeking [59, 60]. Furthermore, studies have shown that NCD patients face stigma and discrimination because of their diagnosis, particularly when the contraction of the disease is perceived to be within the person’s control [61–65]. A study of diabetes-related stigma found that diabetics endure blame, negative social judgment, stereotyping, exclusion, and rejection that have negative behavioral diabetes management implications [62]. Depressed individuals were found, in a qualitative study in Australia, to experience stigma as others believe they are responsible for their own condition [65]. While a qualitative study of breast and cervical cancer prevention and treatment found that participants would take great care to avoid disclosing their cancer diagnosis for fear of isolation, gossip, and poor treatment by their families and communities in part because cancer was believed to be a punishment for bad deeds [61]. Recognizing that NCD patients also face stigmatization is cause for concern as multiple stigmas may be compounded and result in increased marginalization—which has been seen among sex workers and men who have sex with men living with HIV [66] who face both HIV and sex work stigma. It is even possible that NCD stigma could even affect engagement in HIV care continuum and HIV outcomes, through creating additional barriers to care or adversely impacting health and well-being. However, the mechanisms through which stigmas related to belonging to multiple marginalized or stigmatized identities result in health inequalities are not well understood even in the field of HIV [54, 67]. Finally, HIV stigma-reduction strategies in have similarly recommended training all cadres of health facility staff and adopting practices to prevent involuntary disclosure [68, 69].

HIV infrastructure and care strategies are being utilized to meet NCD needs, particularly in low-resource settings. After decades of investment in stopping the HIV epidemic, many systems have been established that can support lifelong chronic care and have been adapted to a variety of settings [17, 18]. As well, decades of perfecting HIV-care delivery and scale-up have resulted in many lessons that can guide the NCD response [16]. While this scenario presents a unique opportunity for developing a sustainable NCD-care response, there are many inherent challenges to using that infrastructure for NCD care in light of HIV stigma. HIV stigma may shape how NCD programs develop, where they are housed, and how they are delivered. This was seen in Kumachek et al.’s qualitative study on integrating HIV testing and cervical cancer screening that recommended integration strategies make an effort to disassociate HIV testing from cervical cancer screening in response to concerns that potential clients would hesitate to come for screening if they believed they might be required to test for HIV [56]. However, recognition that HIV stigma may deter engagement in integrated programs for NCD patients presents an opportunity not to dissociate NCDs and HIV as a means to attract patients, but for HIV-stigma reduction in sero-negative populations. This conundrum of the potential for integration to be both an opportunity for HIV-stigma reduction and a potential barrier to uptake of NCD care warrants further study to better understand and explore the role of stigma in differing models of integrated care.

Findings from this literature point to a need to further understand how HIV stigma may affect NCD care for both PLHIV and non-PLHIV and to expand investment in HIV-stigma reduction. Many of the included articles concluded by recommending such further research and investment [40–42, 44–46, 49–51, 56]. For example, Bynum et al. argue that understanding the ways in which HIV stigma exist and are perpetuated in communities and health care infrastructure can aid efforts to promote and sustain health services utilization [42]. Lessons from the field of HIV-stigma research may provide a means to efficiently gather information on the role of HIV stigma in the NCD-care continuum, guide the development of an effective response, and ultimately support health system strengthening efforts to meet the growing demand for NCD care. For example, research should investigate associations between HIV stigma and clearly defined health care seeking behaviors or outcomes [70] and prioritize understanding the underlying causes or drivers of stigma that can be immediately addressed, such as negative attitudes, fear of infection, or ignorance around the forms that stigma and discrimination take [28, 29] or stigmas unrelated to the condition. Additionally, further research is needed to better understand and address intersectional stigma, particularly as individuals who already may be marginalized not only for their sero- status, but possibly for their race, gender, or sexual orientation, are now facing an elevated risk for joining yet another group stigmatized for health status.

### Limitations

The authors wish to acknowledge several limitations of this scoping review. The abstract screening, full text review, and data charting processes in scoping reviews are inherently subjective. However, efforts to limit subjectivity were made, and each step in the review process was conducted by two researchers with supervisory input from the senior researcher. In addition, this topic has received severely limited attention in the literature. While careful attention was given to assessing the methodological quality of included studies, the researchers aimed to identify any evidence that would provide insight into HIV stigma and NCD care. The results of quality appraisal demonstrated that the quality of included studies varied (Table 2), which may limit the strength of our findings. Additionally, many studies included in this review examined HIV stigma as a secondary or tertiary aim of their analysis. This scarcity of research with a primary aim of investigating HIV stigma and NCD care for PLHIV has implications for the strength of the included evidence. It is possible that other studies similarly recognized or discussed stigma, but did not reference these findings in their abstract and would not have been captured in this review.

## Conclusions

The global burden of NCDs is on the rise, particularly among PLHIV as a result of the nature of HIV and side of effects of its treatment. As health care systems are adapting to meet this epidemiological shift, HIV infrastructure, care strategies, and resources are being leveraged to provide NCD care to both PLHIV and HIV-negative patients. While the detrimental role HIV stigma plays in the HIV-care continuum is well recognized, its potential to deter or disrupt NCD care is not well understood. This literature review suggests that fear of disclosure, internalized shame and embarrassment, and negative past experiences with or perceptions of health care providers negatively influence engagement with NCD care. Furthermore, findings highlight some of the ways HIV stigma can adversely affect not only PHLIV in need of NCD care, but all NCD patients. Some NCDs were shown to be stigmatized in their own right as well because of their association with HIV. Studies included in this review found that integrating NCD and HIV care can both reduce stigma for PLHIV and a present a barrier to access for NCD care. Due to the dearth of available research and the variability in initial findings, further research to explore the role of HIV stigma in the NCD-care continuum for PLHIV is necessary. Lessons from the field of HIV-stigma research can serve as a guide for these efforts.

## Supporting information

S1 File PRISMA checklist. (DOC)

S2 File Search strategy example. (DOCX)
