# Supplementary material for: A scoping review of the role of HIV-related stigma and discrimination in noncommunicable disease care
Source: PLoS One. 2018 Jun 21;13(6):e0199602. doi: 10.1371/journal.pone.0199602 (PMC6013191; doi:10.1371/journal.pone.0199602)
Supplement: S2 File — (DOCX) [file pone.0199602.s002.docx]

Search conducted in Pub Med on May 31, 2017:

Search **(HIV[Title/Abstract] AND (Stigma[Title/Abstract] OR Discrimination[Title/Abstract] OR attitude)[Title/Abstract] AND (noncommunicable[Title/Abstract] OR non communicable[Title/Abstract] OR non-communicable[Title/Abstract] OR cardiovascular[Title/Abstract] OR diabetes[Title/Abstract] OR cancer[Title/Abstract] OR hypertension[Title/Abstract] OR respiratory[Title/Abstract] OR depression[Title/Abstract] OR anxiety)[Title/Abstract])** Sort by: **Best Match** Filters: **Abstract; Publication date from 2007/06/03 to 2017/05/31; English**
